# Exploring Smartphone-Based Edge AI Inferences Using Real Testbeds

**DOI:** 10.3390/s25092875

**Published:** 2025-05-02

**Authors:** Matías Hirsch, Cristian Mateos, Tim A. Majchrzak

**Affiliations:** 1 ISISTAN (UNICEN-CONICET), Tandil 7000, Buenos Aires, Argentina; matias.hirsch@isistan.unicen.edu.ar (M.H.); cristian.mateos@isistan.unicen.edu.ar (C.M.); 2 Faculty of Computer Science, Ruhr University, 44801 Bochum, Germany; 3 Center for Advanced Internet Studies (CAIS), 44801 Bochum, Germany

**Keywords:** edge AI, smartphones, cluster computing, energy efficiency

## Abstract

The increasing availability of lightweight pre-trained models and AI execution frameworks is causing edge AI to become ubiquitous. Particularly, deep learning (DL) models are being used in computer vision (CV) for performing object recognition and image classification tasks in various application domains requiring prompt inferences. Regarding edge AI task execution platforms, some approaches show a strong dependency on cloud resources to complement the computing power offered by local nodes. Other approaches distribute workload horizontally, i.e., by harnessing the power of nearby edge nodes. Many of these efforts experiment with real settings comprising SBC (Single-Board Computer)-like edge nodes only, but few of these consider nomadic hardware such as smartphones. Given the huge popularity of smartphones worldwide and the unlimited scenarios where smartphone clusters could be exploited for providing computing power, this paper sheds some light in answering the following question: Is smartphone-based edge AI a competitive approach for real-time CV inferences? To empirically answer this, we use three pre-trained DL models and eight heterogeneous edge nodes including five low/mid-end smartphones and three SBCs, and compare the performance achieved using workloads from three image stream processing scenarios. Experiments were run with the help of a toolset designed for reproducing battery-driven edge computing tests. We compared latency and energy efficiency achieved by using either several smartphone clusters testbeds or SBCs only. Additionally, for battery-driven settings, we include metrics to measure how workload execution impacts smartphone battery levels. As per the computing capability shown in our experiments, we conclude that edge AI based on smartphone clusters can help in providing valuable resources to contribute to the expansion of edge AI in application scenarios requiring real-time performance.

## 1. Introduction

The application of DL to perform on-site tasks is rapidly expanding. In the healthcare domain, DL is used for predicting, detecting, and diagnosing different diseases [1,2]. In food production, particularly animal farming [3], these tasks include animal behaviour recognition, growth evaluation, and individual identification. In the smart city domain [4,5], tasks include intelligent transportation, waste management, crime prediction, public infrastructure, and green space maintenance. Many of these tasks involve object classification and recognition using images as input. The application of CNNs (Convolutional Neural Networks) has become popular to perform these tasks due to the training procedure requiring minimal human intervention compared to other machine learning techniques. However, training a deep neural network model requires big datasets and, thus, powerful hardware. Nowadays, data gathering leveraged by mobile and IoT devices as well as computing power offered by cloud computing platforms greatly speed up training processes. Cloud computing is considered not only in the training phase of DL models but also for making inferences. It implies that a trained model deployed in a remote computing infrastructure is evaluated with locally generated input data, and produces output results that must be transferred back traversing long-latency internet uplinks and downlinks. This latency has been identified as a limitation for time-sensitive applications. To overcome this issue and others such as the unavailability of an internet connection, network overloading, and privacy protection, edge-based architectures have been proposed [6,7].

There are differences highlighted in the literature when referring to edge-based architectures [6]. Some of these are simply terminological, while others describe substantial differences in the way their constituent components are defined and interoperate with components from other computing layers [8]. Points in common, however, are minimizing data transportation and bringing computing resources close to where computing needs and data originate. Heterogeneity and energy constraints are common features of computing infrastructures operating in cyber–physical and IoT systems. For these reasons, cooperation is a key concept of Dew Computing [9], which is among these edge-based architectures and provides low processing power and consumer devices with the role of main computing resource providers. Among these consumer devices are smartphones. According to [10], nearly 84% of the current world’s population owns a smartphone. Modern smartphones are equipped on average with up to eight cores, powerful GPUs, and several gigabytes of RAM, which are typically underutilized [11], representing underexploited ubiquitous computing cycle reservoirs. A theoretical study [12] estimated that the computing power of 3 million Samsung Galaxy S8 smartphones can be compared to that of 166 thousand servers present in data centers like those owned by Google or Amazon. Other studies [13,14,15] show that carbon-efficient high-performance servers for different purposes, including training complex DNNs, can be built by repurposing old smartphones.

While other works in the field show that AI inferences using heavy DL networks are possible by applying DL model partitioning techniques using SBC clusters connected via wired LANs, in this work, we focus on empirically evaluating the feasibility and effectiveness of wireless collaboration among smartphones for real-time execution of computer vision tasks using DL and applying an intuitive data partition scheme over data stream workloads. The key contributions of this work can be summarized as follows:

- We considered and characterized different workloads associated with distinct application domains with a level of realism that no other study in the field has utilized before. The workloads comprise the usage of three pre-trained DL models capable of being fully loaded and executed in resource-constrained devices for real object recognition and classification, and real input in the form of image sets given in a stream-like fashion emulating different processing needs and application scenarios.
- We comprehensively measured, over real testbeds, the accumulated inference latency and energy consumption that heterogeneous smartphone clusters achieve compared to low-power nodes typically used in edge and IoT applications scenarios, discussing implications and future research necessary to popularize this form of edge computing to application scenarios beyond the illustrative examples shown in this work.
- Load balancing mechanisms heavily affect the performance of HPC systems, with smartphone clusters being no exception to this rule, with the addition that concerns such as battery-related concerns characterize this type of infrastructure. For this reason, we included distinct state-of-the-art load balancing mechanisms in the area in all evaluations associated with smartphone clusters.
- The node and workload setups involved in our experiments represent working examples and a guide on how to tailor and use our experimentation methodology, composed by freely previously published and available tools, specially designed to facilitate the research and experimentation with real smartphone cluster testbeds.

This work is organized as follows. Section 2 presents and classifies recent efforts backed by real testbed results to speed up inferences at the edge using different approaches for distributed execution. Section 3 describes the mobile distributed computing model and infrastructure-related architectural assumptions we made in our exploration tests, as well as details concerning DL models used and data stream workloads. In Section 4, we first explain details about node setups and metrics, and then we present the results obtained in our empirical evaluation. We discuss limitations of our work along with future research directions in Section 5. Lastly, Section 6 presents the conclusions.

## 2. Related Works

In this section, we analyse relevant works in line with the broad concept of *distributed inference*, which is present in recent research related to AI at the network edge. Broadly, in the context of AI, an inference means asking a model to compute a result based on input data (e.g., given an image, list the objects detected). At this point, it is worth differentiating works that employ AI, such as DRL or evolutionary metaheuristics for improving resource utilization at the network edge [16], from works targeting AI task execution, e.g., CV tasks using distributed computing infrastructures at the network edge. In the first group, proposals mostly focus on innovatively modelling variations of NP-hard edge resource allocation problems, while solutions are presented as optimal or near-optimal and compared against others commonly via simulation. In the second group, the focus is on exploiting intrinsic AI tasks characteristics to evaluate different performance metrics of execution schemes that exploit collaboration among distributed nodes, usually exploiting parallel processing opportunities using real testbeds as the evaluation methodology. This section classifies works that belong to the second group. When computing infrastructures of at least two nodes are used to perform inference tasks, it is said that distributed inference is applied. Table 1 shows a first-cut classification of distributed inference schemes, differentiating among vertical [17,18,19,20,21] and horizontal [22,23,24,25,26] ones.

Under a vertical scheme, collaborating nodes typically belong to different computing layers of the edge architecture. These layers are organized in a hierarchical structure where nodes in a layer render services to those in the layer below using services provided by nodes in the layer above. Nodes at the base of such a hierarchy commonly have much less powerful computing capabilities and use the services of layers above to complete computationally intensive tasks. On the other side, nodes at the top layer present more powerful computing capabilities, like fog or cloud resources. However, reaching such nodes results in long-latency communication when transferring task input/output data. For this reason, its usage should be carefully analysed, especially for applications with delay-sensitive time constraints. A classical example of this type of collaboration is given when a resource-limited user device leverages the services of an edge server or edge layer to partially or completely compute the results of inference tasks. Since the edge layer can render computing services to a variable number of users in a locality, depending on the burden at the time of receiving tasks, the edge layer can have enough resources to complete or partially perform a task. In the later case, the edge layer would eventually leverage the services of a remote cloud datacenter to fulfil the request while meeting potential task deadlines. Like in [17,20], offloaded decisions are evaluated considering communication throughput between computing layers. In addition, a task requiring the execution of a DL model is split into several parts. In this splitting process, the authors propose considering insights of data anatomy and computing requirements of layers characterizing different DL models. Partitions of model’s graph are dynamically assigned and executed between a device and remote servers. Other works adopting this distribution scheme are [18,19,21]. Several works provide evidence that vertical collaboration is feasible for allowing resource-limited devices to execute computationally intensive tasks, such as CV tasks using large DL models. However, a major drawback relates to the long-latency communication dependency when offloading tasks to upper resource layers that provide the required computing capability to complete the tasks.

Another inference distribution scheme is when nodes within the same layer collaborate in the execution of computationally intensive tasks. This scheme is known as horizontal collaboration. A key difference with vertical collaboration is that participating nodes and data rarely occur outside the scope of an LAN or WLAN. Even though this form of collaboration governs the internal communication of nodes within the same layer, in this paper, we put special focus on the horizontal communication of resource-constrained nodes, including edge and user device nodes. In the literature, we have identified several works proposing distributed inference under this form of collaboration [22,23,24,25,26]. We further analysed these studies, taking into account several dimensions which were summarized in Table 2: the type of parallelism proposed, i.e., whether it is based on intervening in the anatomy of a DL model, partitioning the data input, or adopting a hybrid approach by combining the previous techniques. Another dimension of analysis relates to the methodology used to evaluate the proposal. We selected works whose methodology involved setting up real testbeds and we describe the hardware used and the way resource heterogeneity is present in the experiments. Moreover, with it being provided that QoS is a concept that relates to adapting to dynamic changes in the workload and/or available resources, we indicate the support of these works in this respect. Finally, we contextualize the strategy of each work for resource management, mentioning the main motivation behind the proposal.

Works can be also classified according to how workload execution is parallelized. We found three approaches of parallelism: model partitioning, data partitioning, and hybrid. Within the former group, layers composing a DL model are deployed into different devices so that outputs computed by a device are the inputs for another one. With this type of parallelism, all participating devices are involved in computing inferences for all portions of the input. By contrast, when using data partitioning parallelism, edge nodes run the whole model over portions of the input. A third type, which we call hybrid, parallelizes workload by combining the data and model partitioning schemes. In all cases, results for the whole input are obtained by joining partial results calculated by all participating devices.

When several devices participate in inferencing tasks, two device-related questions emerge: Does the approach consider executing inferences on heterogeneous devices? If so, what does the approach do to deal with the performance differences due to executing the same workload on devices with different computing capabilities to ensure certain QoS levels?

In [22], the authors propose AutoDiCE, a tool for partitioning, deploying, and distributively executing a large CNN model on multiple resource-constrained edge devices. The platform specification and mapping specification allow practitioners to indicate which sub-model runs on which edge devices. Moreover, once generated and deployed, mappings between sub-model and edge nodes are fixed at runtime. Communication between sub-models and resources usage is implemented via the well-known parallel programming standards MPI and OpenMP. Although the tool was designed to support horizontally and vertically distributed inferences, it was tested using the former scheme, using an infrastructure comprising homogeneous edge nodes communicated via a gigabit Ethernet switch. Particularly, they use different LAN settings with up to eight edge devices (Nvidia Jetson Xavier NX). DeColla [23] utilizes Deep Reinforcement Learning (DRL)-based QoS adaptive allocation for horizontal collaborative inference, complemented with a fault tolerance mechanism implemented using a message queue. All DL layers are present in all collaborating IoT devices. Neuron calculation in each layer occurs in parallel, coordinated via a message queue and orchestrated by the DeColla engine, which operates in a requester IoT device. The number of IoT devices, available computing resources, and the network state are evaluated online upon each neuron assignment. The experimental setting was an LAN with three Raspberry Pi 4 models.

In [24], the most appropriate version among different pruned models is selected at runtime via an adaptive workload distribution to meet the required application-specific accuracy and performance level. Such dynamic selection is performed using base knowledge of accuracy–performance tradeoffs profiled from heterogeneous edge nodes, together with a runtime monitoring system for resource management. Evaluation was performed over an LAN with four IoT devices (2x Odroid XU4, Jetson Nano, Rapsberry Pi 4). In [25], the DeepThings framework for executing large CNN models in resource-constrained devices was proposed. The framework adopts a hybrid parallelization approach due to its combining model and data partitioning schemes. On one side, as an offline step, the framework fuses convolutional and pooling layers to create independent and distributable execution units, which leads to reduced memory footprint and allows resource-limited devices to contribute via the computation of partial results that are combined to produce the final result of an inference task. In addition, the workload, represented by a single frame, is divided into overlapping and distributable regions that are computed collaboratively by a group of edge devices. With this scheme, inference capability differences of participating devices are handled with two strategies. One of these is static and represented by the fact that memory requirements of fused layers executed by a device adapt to its available memory resources. The other strategy is that data regions are dynamically distributed according to device computing capabilities by following a work stealing scheme. The experimental setup comprises a WLAN with up to six IoT devices (Raspberry Pi 3). Another work adopting hybrid parallelization is eDDNN [26], which leverages cross-platform web technologies and the WebRTC protocol to allow for distributed inference among heterogeneous end devices (smartphones). The collaboration is mediated by an edge server that keeps track of node and task status information. Tasks, determined by images input size, and the DL model to be used, are divided into sub-images and sub-models by a decision-maker component that coordinates the execution of different pieces among end devices using a shared dependency table and dynamic node information. Edge data duplication is present at the step of dividing an image into sub-images. When distributing a convolutional operation by dividing the input and the model, duplication is required for ensuring the correctness of results. To evaluate the approach, they mostly rely on simulation, with data profiled from hardware running real workloads, and they also include some experiments with real settings.

In the discussed works, horizontal distributed inference based on the collaboration of battery-operated end-user devices, such as smartphones, combined with data partitioning schemes is rare, meaning that the benefits and limitations of performing real-time CV tasks—and possibly other AI tasks as well—are as yet unknown. Our work sheds light on providing empirical evidence for quantifying the computing power of smartphone clusters. Existing approaches exploiting data partitioning schemes add redundant data to the original input for performing distributed inference. We propose a simple yet effective adaptive data partitioning scheme for performing real-time CV tasks, which works under a pull-based scheme where tasks are extracted by devices from a shared queue. This is evaluated under varying settings of real smartphones and SBCs cooperating in WLAN clusters. Since performance may vary according to how workload is distributed, we provide insights on the performance achieved and energy-related measurements using different state-of-the-art online heuristics and smartphone clusters. Moreover, performance comparisons of different <smartphone clusters, load balancing heuristics> combinations are compared against the performance achieved by different edge nodes commonly used in IoT applications. All experiments performed in this work demonstrate the viability of constructing a platform for in-vivo experiments whose main components have been previously published, validated, and made publicly available for use [27,28,29].

## 3. System Model and Assumptions

We envision a system that opportunistically scavenges computing cycles of nomadic hardware, such as smartphones, to complement—or eventually replace—the computing cycles delivered by low-power edge nodes. Particularly, we shed light on the computing capability of different smartphone clusters and load balancing heuristics for processing streams at the edge. In this work, we consider streams of images analysed using CV tasks in real-time or near real-time by harnessing deep neural networks. Defining appropriate incentives for smartphone users—tasks executors, privacy preservation, and security mechanisms for task submitters and task executors—is outside the scope of the current work [30,31,32].

**Figure 1:**
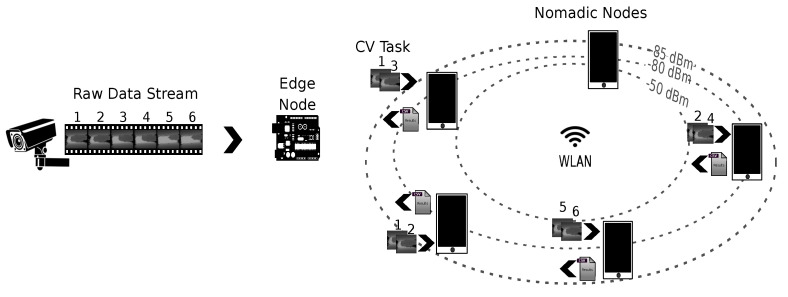
Schematic overview of main nodes.

We assume a master–worker architecture operating within the scope of WLAN, with a master node officiating the tasks performed by submitter and worker nodes contributing with computing resources, i.e., playing the role of tasks executors (see Figure 1). A master node is assigned the roles of data concentrator and task coordinator. Playing the first role implies that the node exposes end points for receiving raw data and preprocessed data. On one side, raw data transferred to a master node are captured by devices with sensing capabilities, e.g., RGB, IR, or depth images taken with different camera devices. On the other side, pre-processed data are obtained by applying a series of computational operations to decode, synthesize, and/or represent raw data in a format that can be more efficiently stored, retrieved, or analyzed. Moreover, playing the role of task coordinator means that the node runs logic to decide how to distribute the load involved in preprocessing raw data by available worker nodes to meet the expected QoS. Finally, worker nodes opportunistically connected to the WLAN register through a master node service to contribute with computing resources and wait for tasks assigned by the master node. As task completion occurs at the worker nodes, results are sent back to the master node.

With this architecture, we aim to measure the feasibility of using different smartphone groups cooperating under the same WLAN for performing CV tasks, and compare the performance achieved with that of SBC-like edge nodes commonly used for servicing IoT and edge applications.

### 3.1. Empirical Evaluation

We employed different hardware for the edge nodes, whose characteristics are shown in Table 3. All nodes have multicore processors. All nodes but the Nvidia Jetson Nano have 802.11 WiFi radio, with a theoretical bandwidth capacity between hundreds of Mbps (norm n) to a few Gbps (norm ac). In fact, SBC node WiFi was not used because, in our experiments, these nodes play all the roles—task submitter, task coordinator, and task executor—simultaneously and no data transfers are involved. The main memory of the edge nodes ranges from 2 to 8 GB. In our setups, SBCs operate plugged into the electricity power grid, while smartphones operate using their battery as the main power source. Additionally, we include an estimated per unit acquisition cost in USD. When searching for cost-effective configurations, the cost can be used as a deciding factor in situations where several node combinations achieved the desired performance.

Section 3.2 elaborates on the deep learning model characteristics considered in the experiments. In Section 3.3, we describe three study cases belonging to different application domains, how image streams are produced, and task characteristics. Finally, Section 4.1 presents the comparison results based on the designed benchmarks, which we explain in the following sections.

### 3.2. DL Models Benchmarking

Benchmarking is a relevant task to approximate node performance in solving a specific task and this procedure was carried out for nodes in Table 3, where tasks refer to the making of inferences using different Tensorflow DL models. Tensorflow is a popular library for creating, training, evaluating, and executing deep learning models. The Tensorflow project [33] offers a benchmarking tool to collect statistical information of a.tflite model, one of the file formats for created models. The tool measures the time a model takes to produce an inference and the memory consumed, among other information using randomly generated synthetic model input. It is worth mentioning that all inference times reported in this paper are on the basis of using CPU support. Even though, today, there is specialized hardware—sometimes referred to as AI hardware accelerators—to run AI logic faster than with general purpose hardware like CPUs, there are some practical limitations, including library support for different platforms and proprietary chips embedded only in high-end smartphones of certain brands, which still prevent the massive use of mobile devices for the distributed computing we have in mind.

**Figure 2:**
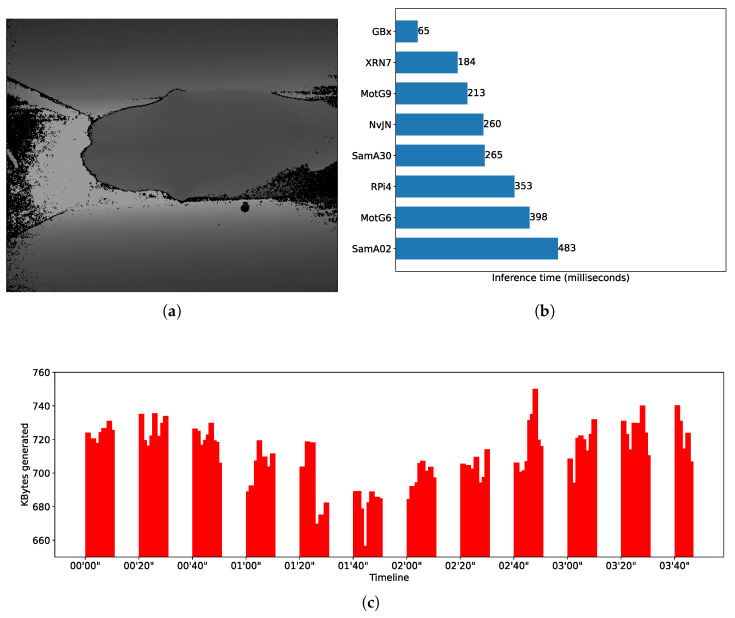
BCS—workload characterization. (**a**) Input image example taken using the Kinect v2 depth camera. For reference, the image partially shows the cow’s crest/neck on the right, the rump/tail head on the left, and the walls of the runway to the milking parlor on the top and the bottom. (**b**) SqueezeNet model benchmarking. Numbers represent inference time per depth image in milliseconds. (**c**) BCS data stream excerpt.

**Figure 3:**
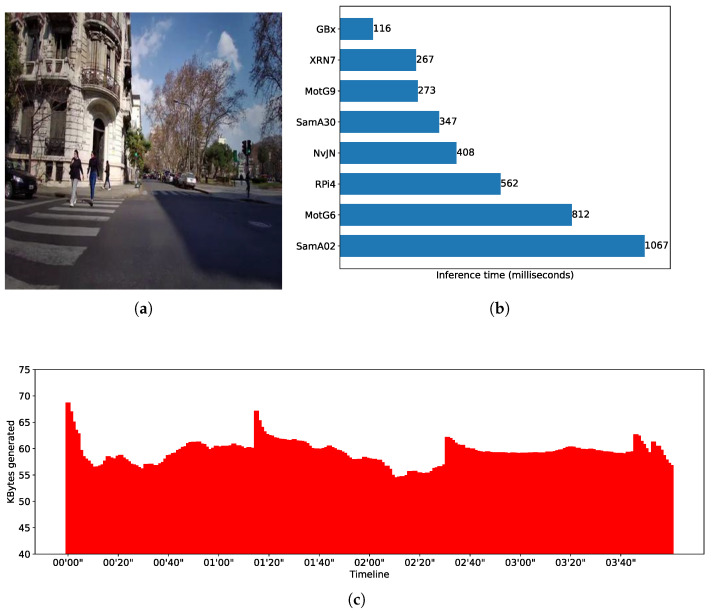
SWT—workload characterization. (**a**) Input image example. (**b**) YoloV4 tiny model benchmarking. Numbers represent inference time per image in milliseconds. (**c**) SWT data stream excerpt.

**Figure 4:**
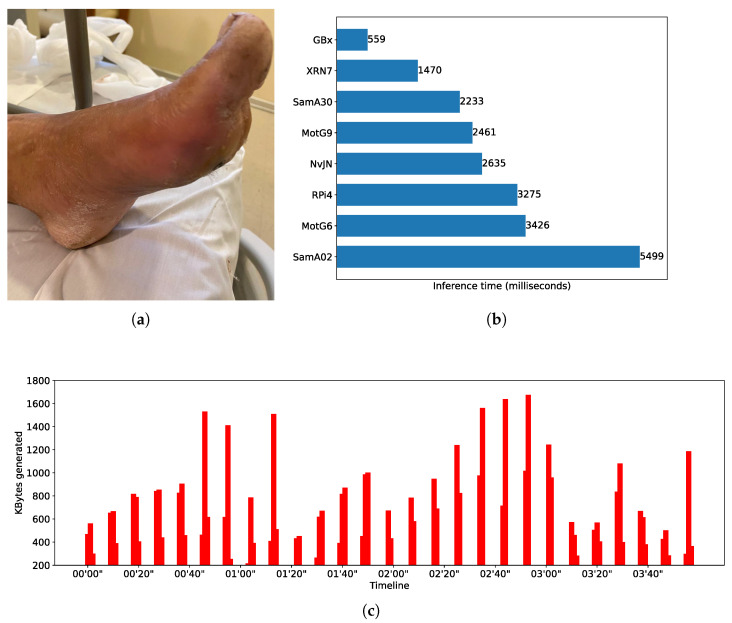
DMED—workload characterization. (**a**) Input image example. (**b**) EfficientNetB4 model benchmarking. Numbers represent inference time per image in milliseconds. (**c**) DMED data stream excerpt.

Moreover, in our study, the notion of *real-time* is context-dependent. Depending on the application, a constant delay of a few seconds might satisfy the notion of real-time. For this reason and with the aim of evaluating smartphone-based settings in different application scenarios, i.e., with different real-time requirements, we used three DL models that present quite dissimilar inference times. It is not part of the evaluation core to test DL models in their most efficient version—i.e., we do not aim to test and improve model performance per se. Rather, our objective is to explore the feasibility of using smartphone-based opportunistic clusters as alternative edge devices for completing resource-intensive AI workloads of different characteristics. To introduce variability in the handled workloads, we chose three DL models that could be fully loaded using resources (e.g., RAM space) present in devices like smartphones and SBCs; this resulted in quite dissimilar inference times, which can be seen from Figure 2b, Figure 3b and Figure 4b. In addition, the three models were subject to distinct image generation rates depending on the needs of the application domain. In addition, given that these models were conceived to be used for different CV tasks, their selection allowed us to tailor feasibility tests targeting different application domains, which is a research gap in the field of study.

All models used in the tests are pre-trained object recognition models that take images as input and produce text as output. Text refers to bounding boxes, classes, and confidence levels of recognized objects, or categories of a unique object depending on the purpose the model was trained for. To test different mobile distributed computing scenarios, we intentionally selected models that, when used to make inferences, present dissimilar computing times. One of the models is YoloV4 [34], which is used to perform real-time object detection. Figure 3b shows average inference times (in milliseconds) for different nodes when performing object detection using a YoloV4-tiny model, which is the version that can be run on mobile devices using Tensorflow. We see, for instance, that a smartphone Xiaomi RN7 makes inferences in around 248 milliseconds—i.e., it is able to process up to 4 FPS, doubling the capability of a RaspberryPi4—but it is slower than a Gigabyte Brix, which reaches nearly 10 FPS. Another of the benchmarked models encapsulates expert knowledge of what is known as the Body Condition Score (BCS) [35] and was proposed to score dairy cows to differentiate healthy and non-healthy cows. The model uses SqueezeNet as its base neural network. Figure 2b shows the BCS average inference time for different edge nodes. For instance, the Xiaomi RN7 smartphone completes an inference in around 184 milliseconds; i.e., it is able to deliver barely more than 5 FPS, while a Gigabyte Brix node is around 15 FPS. The third model used is based on an EfficientNetB4 and was trained to recognize progression of diabetes using foot images as input [36]. Figure 4b shows the inferences times obtained by different edge nodes. By comparing the fastest smartphone with the fastest SBC, it can be noted that Xiaomi RN7 completes an inference in around 1470 milliseconds while the Gigabyte Brix does the same in approximately 559 milliseconds. When comparing performance of DL models, notice that for most of the edge node benchmarks, inference times differ up to one order of magnitude. As said, the Xiaomi RN7 completes inferences in 184, 248, and 1470 milliseconds for the SqueezeNet, the YoloV4, and the EfficientNetB4 models, respectively.

### 3.3. Workload Characterization

In this section, we describe the workloads associated with the machine vision tasks that were performed over stream-like inputs. Indeed, machine vision tasks such as object detection and classification using CNNs are commonly applied in diverse domains. To complete the experimental setting, we consider video frame streams of different lengths and distinct frame rates, which serve as input to the CNN models described above and, in conjunction, both element0s shape the heterogeneous workloads of the mobile distributed computing setups under evaluation.

Without losing generality, we assign each model–stream pair different domain/usage contexts. These domains are a dairy farm application scenario called “Body Condition Score” (BCS), a smart city application scenario that we call “Sense while travel” (SWT), and a human healthcare monitoring application scenario called “Disease Monitoring and Early Diagnosis” (DMED). Next, we describe the dynamics of each scenarioin order to explain to the relevant stream-like input:

- Body Condition Score (BCS): In a dairy farm, outfitted cows—i.e., cows that are either overweight or underweight—tend to produce less milk and can breed less frequently than those properly weighted. Identifying such animals so as to give them proper treatment is crucial to maintain the full productivity of cows. To detect such animals, a CNN model is used, which takes depth images taken from the top with a camera strategically positioned as cows walk to a milking parlor [35]. The image capturing procedure is performed within a time window that does not exceed 10 s. For the model to produce accurate results, it is important that the animals do not stand directly under the camera and that the captured images contain a specific part of the cow; the result is that a high percentage of captured images, say 50%, is dropped, i.e., tagged and filtered, as these images do not represent useful input for the CNN model. Moreover, during the transition of one animal to the next under the lens of the camera, which can take 10 s on average, it is reasonable to stop capturing images. To make the body condition score calculus feasible, reliable, and energy efficient, no less than one hundred frames are required to serve as input for the CNN model, for each cow. All these constraints configure a stream processing scenario where depth images can be produced at a rate of 15 FPS during 10 s, followed by another 10 s without image capture. We decided that an atomic CV task would be represented by the burden of applying the CNN model to fifteen consecutive frames, meaning that a CV task is created every second, which gives a total of ten CV tasks created for a cow. Considering that the dataset used to recreate this scenario includes 1740 images, the resulting workload stream comprises 116 CV tasks given within a time window of 3 min and 34 s.
- Sense while Travel (SWT): A city can take advantage of vehicles such as urban line buses to collect information about relevant street events while travelling, e.g., for statistical purposes, crime detection, and infrastructure maintenance planning. Images captured with a bus front camera might feed a YOLO model, which in turn gives a plain text representation of objects present in an image and the accuracy percentage of each detected object class as output. An energy efficient way to achieve this without having to process a large number of frames with redundant information is to cleverly select a sample rate according to the expected moving change of detection targets. We landed on a sample rate of 2 FPS and evaluate the system performance for a stream duration of 30 min. These parameters mean sensing data for 15 km assuming a vehicle travelling the road at 30 km/h. A CV task is represented by the execution of the CNN model on two consecutive filtered frames, meaning that a CV task is created every second. Considering that the dataset used to recreate this scenario includes 3600 images, the resulting workload stream comprises 1800 CV tasks given within a time window of 30 min.
- Disease Monitoring and Early Diagnosis (DMED): We envision a remote monitoring mobile health scenario where a CNN model encapsulates expert knowledge on different pathologies to assist people, especially older people, receiving intensive care services; the scenario also involves analysing different health parameters as a way to prevent certain pathologies and monitor pre-existing illnesses. We consider a scenario where a caregiver or nurse rendering services in an elderly residence is assigned with the task of taking foot tissue pictures for all patients, resulting in a data stream of 10 FPS during 2 s, with pauses of 7 s without capturing images. In this case, a CV task executes the model described in [36] to two consecutive frames, meaning that during the image capturing time, five CV tasks are created per second. Considering that the dataset used to recreate this scenario includes 600 images, the resulting workload stream comprises 300 CV tasks given within a time window of 8 min and 55 s.

Figure 2a,c show a timeline representation of the data input stream shape, i.e., the kilobytes of data transferred per second, and an example of the input for the CNN used in the Body Condition Score scenario. Similar information can be found in Figure 3a,c and Figure 4a,c for the SWT and DMED scenarios, respectively. In all cases, we depict the first four minutes of the three image streams, and we report per-image inference times.

Irrespective of the application domain, these example application scenarios present streams with different characteristics due not only to the dynamics of input generation but also to the computing capability required to perform inferences over the input. In addition, it is assumed that inference results should be available at the master node with minimum latency, i.e., as close as possible to real-time.

## 4. Experiments Setup and Metrics

For setting up and executing tests, we employed a tool set combining several open-source programs [27,28] and hardware [29] modules. The tool set allowed us to automate tasks involved in experimenting with smartphones and real workloads including cluster formation, device preparation (battery charge/discharge actions), deep learning model deployment, stream dynamics reproduction, and result collection and summarization.

Algorithm 1 details all steps involved in running the experiments over real testbeds using different tools:

*Step 1* refers to the initialization of the Motrol hardware used to control battery charging/discharging periods of the devices attached to it. Since charging/discharging actions associated with Motrol connection slots can be triggered wirelessly via HTTP messages sent to a server running inside the Motrol hardware, the hardware setup includes the configuration of the WiFi network (SSID and password) where the server uses a REST API and listens for messages to enable/disable current to each slot.

*Step 2* refers to a phase where devices that need to be controlled are attached to Motrol slots. In this phase, devices are also installed with the software that loads the corresponding DL model associated with each specific evaluation scenario.

*Step 3* refers to the creation of files describing the workload dynamics and granularity, cluster information, including participating devices identified by the smartphone model, the battery level each device must have as precondition to start a test, and the load balancing mechanism to be used to distribute workload. These files must be placed inside a directory named “scn” within the LiveDewStream tool.

*Step 4* is optional and involves the utilization of BAGESS software [28] to define an execution sequence for all scenarios built in Step 3. The derived sequence reduces the execution of all scenarios by optimizing the preparation time of all devices between the execution of one scenario and the next one, while reducing the battery stress of all devices produced as a consequence of all charging/discharging actions triggered by different workload scenarios.

*Step 5* is when all workload scenarios are executed. This involves Step 5.1, when smartphone cluster formation takes place—i.e., devices attached to Motrol are considered or not as part of the cluster, which causes their batteries to be charged or discharged—prepared—to meet the initial battery level required by the scenario under execution. Moreover, Step 5.2 describes the moment when all participating devices are ready to start the scenario execution and it is effectively run until the workload described in the scenario descriptor file is completed. Lastly, Step 5.3 is triggered when the execution of a scenario finishes and generated log files and workload results are moved to a safe directory in the file system to prevent a new scenario execution from overriding the generated data.

*Step 6* is when logs and result files are processed with scripts to create reports and facilitate the analysis of different metrics.

As discussed earlier in this paper, workloads in edge computing and IoT scenarios are commonly computed with medium-to-high-end edge nodes such as SBCs. In the benchmarks, we compare the performance of several SBC-based and smartphone-based setups to complete the workloads described in Section 3.3. The “Edge Setup” column in Table A1, Table A2 and Table A3 differentiates both setups. Considering that smartphone-based setups comprise clusters of smartphones, Table 4 expands on the model and battery information of nodes integrating each cluster. All clusters include a single instance of each smartphone model. Smartphone brands and models are indicated using a short name that can be found in Table 3. The order in which these short names appear in a row corresponds to the order in which smartphones’ initial battery values appear. The latter were randomly chosen. Cluster heterogeneity arises from the combination of smartphone quantities, models, and initial battery levels. Cluster maximum energy and cluster initial energy, both expressed in Joules, in addition to cluster initial battery, which is expressed in %, all derive from individual smartphones data and complement the cluster information.
**Algorithm 1** Clusters Management Overview: Steps involved in setting up nodes and workloads, running scenarios, and deriving metrics using Motrol [29], LiveDewStream [27], and BAGESS [28] toolsStep 1: Setup and initiate Motrol hardwareStep 2: Register $DEVICES to MotrolStep 3: Build $SCENARIO_DESCRIPTOR_LIST to feed LiveDewStream toolStep 4 (optional): Sort $SCENARIO_DESCRIPTOR_LIST  elements by using BAGESS tool to optimize scenarios runtime executionStep 5: For each element in $SCENARIO_DESCRIPTOR_LIST:       5.1 Form Cluster and prepare $DEVICES       5.2 Run scenario       5.3 Collect resultsStep 6: Summarize results to generate metrics

**Figure 5:**
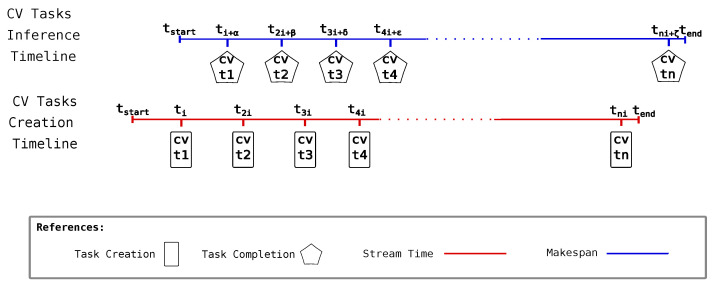
Accumulated inference latency: visual explanation.

One of the metrics we report is accumulated inference latency, a measure of how long the makespan deviates from the stream time. The stream time is the time window where CV tasks are created, while makespan is the time the inference system employs in completing all the created CV tasks. Figure 5 graphically represents how these entities coexist in time. Inference completion of a task naturally occurs in the time after the CV task creation. While task creation can occur by following a quite controlled and periodic process, the task inference process cannot be so controlled. This is what α,β,γ,δ,ε, and ζ represent in the CV task inference timeline associated with each CV task completion event—i.e., different completion times could be given as a consequence of completion, but they are not limited to variations in network latency, CNN data input content, and node execution capability. When subtracting stream time from makespan, the result is the accumulated inference latency. The closer this difference is to zero, the better the setup capability is at responding with proper inference times compared to the time taken to create all CV tasks. A derived metric that gives a notion of the average time employed by the setup to complete a CV task after a new CV task is available could be obtained by dividing the accumulated inference latency by the number of CV tasks created. We call this the inference latency.

**Figure 6:**
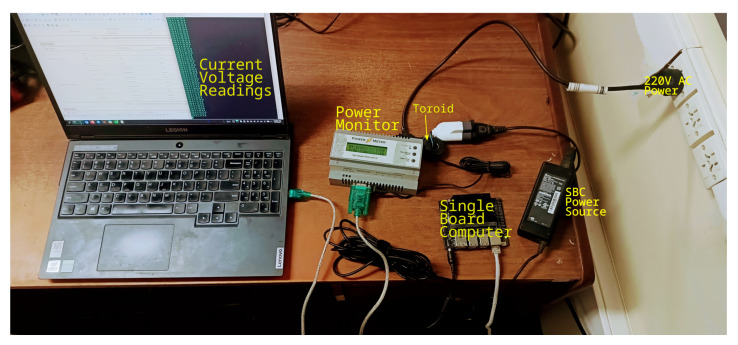
SBC power monitoring connection scheme.

Another metric we report is total energy consumption. To measure energy consumption, we followed two approaches depending on the type of setup. For SBC-based setups, we employed a power monitor [37] that used a toroid connected as shown in Figure 6, which allowed us to collect current and voltage readings from an SBC in a non-invasive way while running workloads. The power monitor was connected to the 220 V AC power and it had a socket after a toroid where the SBC power source was connected. Current and voltage readings were transmitted in real-time via a USB port to a laptop. The power monitor gave two current and voltage values per second. Then, for a workload scenario whose inference time was around 3 min and 50 s, the reported energy consumption was obtained by averaging 460 samples.

For smartphone-based setups, the described approach for registering energy consumption cannot be used, because smartphone batteries are employed as the power source. We thus followed a different approach by registering smartphones battery levels at the beginning and at the end of a workload scenario. Then, we used battery manufacturer information—such as battery mAh and operating voltage—for each smartphone to approximate the total energy used during the workload scenario execution (WSE), expressed in Joules:

(1) $$WSEJoules=\sum_{i=0}^{\#smartphones}\left(battLevelDrops^{i}\times battMAh^{i}\times 0.01\right)\times battVoltage^{i}\times 3.6$$

where #smartphones corresponds to the amount of smartphones integrating the cluster, and $battLevelDrops^{i}$ is the amount of battery level drops registered for the smartphone *i* used for executing the assigned workload, which is in turn calculated as

(2) $$battLevelDrops=batt_{endWSE}-batt_{startWSE}$$

where $batt_{startWSE}$ and $batt_{endWSE}$ are the battery levels of smartphone *i* at the start and end of the workload scenario execution, respectively, and $battMah^{i}$ and $battVoltage^{i}$ are milli-ampere hours and operating voltage information, respectively, provided by the battery manufacturer of smartphone *i*. The constant 0.01 is used as an alternative form of expressing the algebraic operation of dividing a value x by 100. For the case of (1), such an operation is involved in the application of the direct rule of three to express a battery percentage in mAh. Formula (1) emerges from combining a rule of three that relates battery percentage usage with the formula mAh×voltage×3.6=JoulesOfenergy, used to convert mAh to Joules, where 3.6 is a conversion factor that arises from converting milli-ampere hours (mAh) to Coulombs—e.g., 1 mAh = 1/1000 Amperes ∗ 60 s ∗ 60 min = 3.6 Coulombs

Additionally, since a workload scenario execution using smartphone-based setups might cause different battery percentage usage in each node, we report Jain’s fairness index [38] as a metric that summarizes intra-cluster energy utilization. In other words, the metric is used to measure the disparity of energy pulled by the system from resource provider nodes. The metric takes values from 0 to 1. The nearer the index to the value of one, the more balanced the energy utilization among cluster nodes is. The Jain’s index inspired formula we applied is

(3) $$Fairness=\frac{{\left[\sum_{i=0}^{\#smartphones}battLevelDrops^{i}\right]}^{2}}{\#smartphones\times \left(\sum_{i=0}^{\#smartphones}{\left[(battLevelDrops^{i})\right]}^{2}\right)}$$

Since all smartphone-based setups comprise clusters of at least two heterogeneous smartphones, it was necessary to indicate the load balancing strategy used to distribute the load between these. For this reason, all smartphone-based setups were indicated with Round Robin, ComTECAC, or Pull-Based in the load balancing column. Round Robin is a classic load balancing strategy that assigns an incoming task to the next available node using a rotating scheme, ensuring workload is evenly distributed among all participating devices. ComTECAC is a heuristic that captures unique characteristics of heterogeneous smartphone clusters. It is used to rank candidate nodes based on a criterion that combines computation–communication throughput and energy contribution data. It was considered because, as shown in [39], it achieves the best performance compared to other state-of-the-art heuristics. Lastly, with a Pull-Based approach, tasks are pulled by nodes from a shared queue based on their availability. In our configurations, cluster nodes were configured to pull at most one task per request. In turn, a node is able to make a request when the result of the previously pulled task has been sent back to the edge master—i.e., the task is marked by the system as completed.

### 4.1. Benchmark Results

#### 4.1.1. Performance Analysis

**Figure 7:**
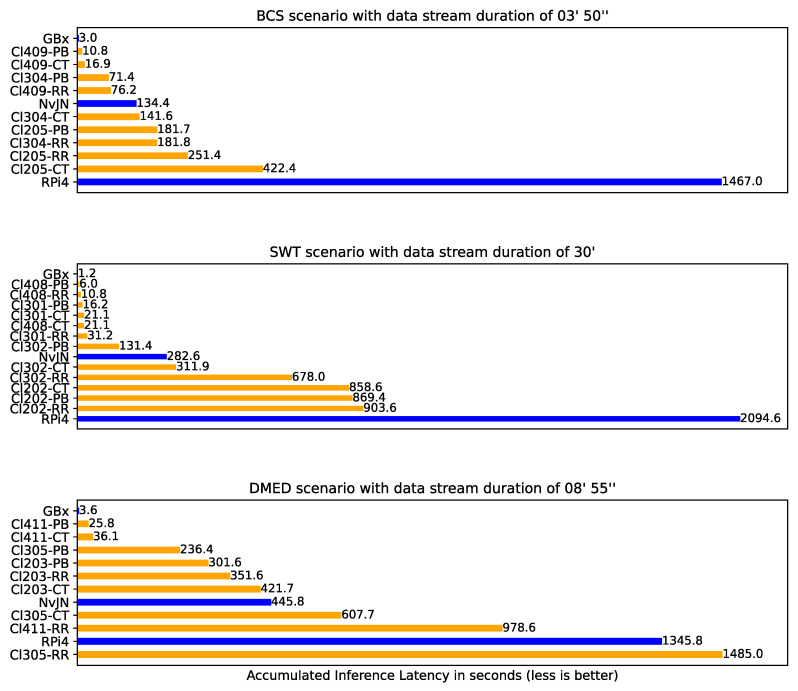
Accumulated inference latency (in seconds) for the three scenarios. For the cluster setups, we indicate the load balancing technique used (PB = Pull-Based; CT = ComTECAC; RR = Round Robin). Load balancing is not used in SBC-based setups.

Figure 7 shows the performance achieved by all setups for all scenarios. Blue bars correspond to SBC-based setups while orange ones correspond to smartphone-based setups. By comparing all setups, it can be clearly noted that the best performance, i.e., the lowest accumulated inference latency value in all tested scenarios, is achieved with the Gigabyte Brix setup. The close-to-zero value indicates that inferences performed with this setup are near real-time—i.e., inference results associated with all CV tasks are obtained within the stream time. Concretely, in the BCS scenario, the accumulated latency in a stream time of $3^{\prime }50^{\prime \prime }$ was 3 s. In the SWT scenario, the accumulated latency in a stream time of $30^{\prime }$ was 1.2 s, while in the DMED scenario with a stream time of $8^{\prime }55^{\prime \prime }$, the value was 3.6 s. With the Raspberry Pi4 and Nvidia Jetson Nano setups, the obtained accumulated inference latency was significantly greater, and in most cases, the inference latency was the stream time multiplied by a factor of two to four—for detailed values, please refer to tables in Appendix A. The Nvidia Jetson Nano setups performed better than the Raspberry Pi4 setups in all cases. However, the workload always exceeded the inference capability. With these setups, reducing workload would be necessary to achieve near real-time performance. Some alternatives to reducing workload are creating more lightweight CV tasks by dropping frames and/or offloading some CV tasks to other edge nodes. These alternatives could come in turn with some QoS degradation as a result of increased network latency.

When comparing smartphone-based setups for the BCS scenario, we empirically confirm what other studies have found [26,40]: by incrementing the number of participating smartphones, performance improves—i.e, lower accumulated inference latency is obtained. This is also true for the SWT and DMED scenarios. However, not only the number of smartphones impacts the setup inference service capability, but also the smartphone computing characteristics. Clusters 301 and 302, for instance, are of the same size. Both clusters are composed by the Samsung A30 and the Motorola Moto G9 Play. However, the Samsung A02 smartphone present in Cluster 302 is not present in Cluster 301, which has the Xiaomi Redmi Note 7 instead. If we compare the inference times associated with the latter mentioned smartphone models and those reported in Figure 2b, Figure 3b, and Figure 4b, we note that the computing capability of the Samsung A02 is between 2.62 and 3.99 times slower than that of the Xiaomi Redmi Note 7. The effect of this difference is observed in the performance values registered for the SWT scenario, where both clusters are tested. While the accumulated inference latency with Cluster 301 was between ∽16 and ∽31 s, for Cluster 302 it was between ∽131 and ∽678 s.

Another observation when comparing smartphone-based setups is that, in general, the Pull-Based load balancing scheme achieves lower accumulated inference latency than Round Robin and ComTECAC. This is because using Pull-Based scheme allows the cluster to balance the workload in accordance with the availability and computing capability of worker nodes rather than treat all nodes with the same capability, as done by Round Robin. The advantage of Pull-Based over Round Robin diminishes in clusters where nodes present quite similar computing capabilities, which is the case for Cluster 202 in the SWT scenario, or even when computing resources are apparently abundant enough to satisfy the workload requirements, which seems to be the case for Cluster 408. The differentiation of node capabilities is also a feature of ComTECAC, which, in most cases, performs slightly worse than Pull-Based but better than Round Robin. In general, the most competitive accumulated inference latency comparison to that obtained by the Gigabyte Brix setup was always achieved with the Pull-Based load balancing scheme. For example, in the BCS scenario, the difference between the Cluster 409 and Gigabyte Brix setups was less than 8 s. The third-best performance was also achieved with a Pull-Based setup. The Cl304-PB, with a size 3 cluster, achieves quite similar performance to that of a size 4 cluster combined with Round Robin, i.e., Cl409-RR. A similar analysis is valid in the SWT and DMED scenarios, with Clusters 408 and 411, respectively. The Pull-Based load balancing scheme achieves considerably better performance than Round Robin and also slightly better performance than ComTECAC, especially when cluster nodes present heterogeneous computing capabilities or when available computing capabilities are tight for coping with real-time tasks requirements.

#### 4.1.2. Energy Consumption–Performance Tradeoff Analysis

**Figure 8:**
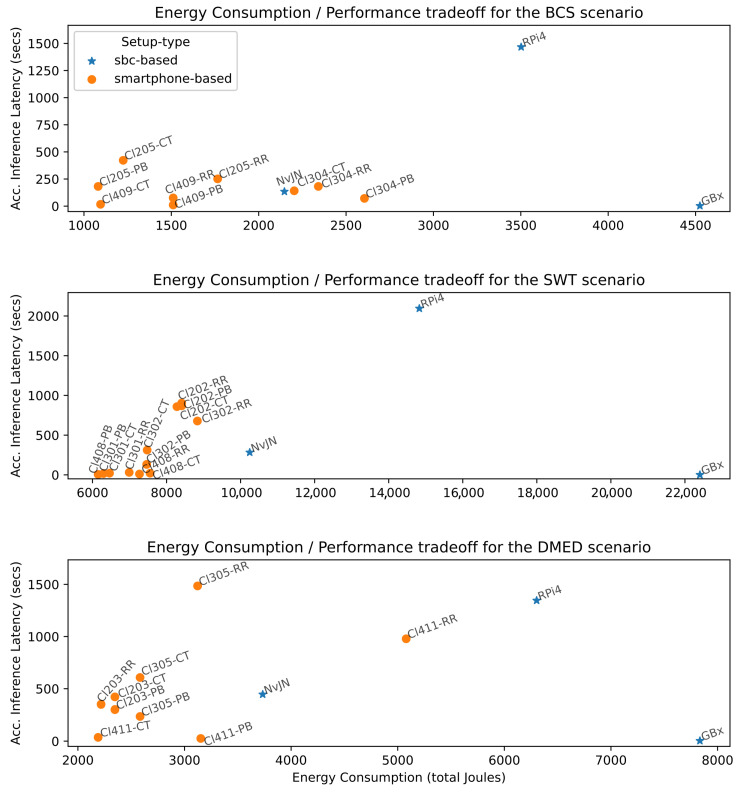
Energy consumption/performance tradeoff.

In view of the high energy consumption that AI applications already demand and expected further increases, studies in this regard [41,42,43] and initiatives such as the Low-Power Recognition Challenge [44] encourage the improvement of not only DL model performance but also the energy that execution and training consume. Thus, we study the tradeoff between performance and energy consumed in all evaluated scenarios. Figure 8 reports the energy consumption and accumulated inference latency tradeoff between different setups in each tested scenario. The best setups are those that achieve the minimum accumulated inference latency using the least energy, i.e., those positioned close to the origin. As can be observed, in all scenarios, the best balance is always achieved by orange dots with a label starting with Cl, which corresponds to smartphone-based setups. It can be noted, for instance, that the accumulated inference latency of GBx setups are the lowest in BCS, SWT, and DMED scenarios; however, these are also associated with the highest values of energy utilization—4524, 22,403, and 7834 Joules, respectively. Interestingly, smartphone-based setups, particularly four-node clusters using Pull-Based and ComTECAC load balancing schemes, offer a good tradeoff in all scenarios, i.e., competitive performance with reduced energy consumption. Some examples are Cl409-PB, Cl408-PB, and Cl411-PB for the BCS, SWT, and DMED scenarios, respectively. Also, Cl409-CT and Cl411-CT for BCS and DMED, respectively. In other words, for the BCS scenario, when it is acceptable to have all CV task results calculated with a delay of 7.8 s w.r.t to the fastest setup—GBx—Cluster 409 with a Pull-Based scheme can achieve the objective by utilizing ∽1510 Joules, i.e., almost three times less energy than the Gigabyte Brix setup. Similar relations can be found for the SWT and DMED scenarios.

**Figure 9:**
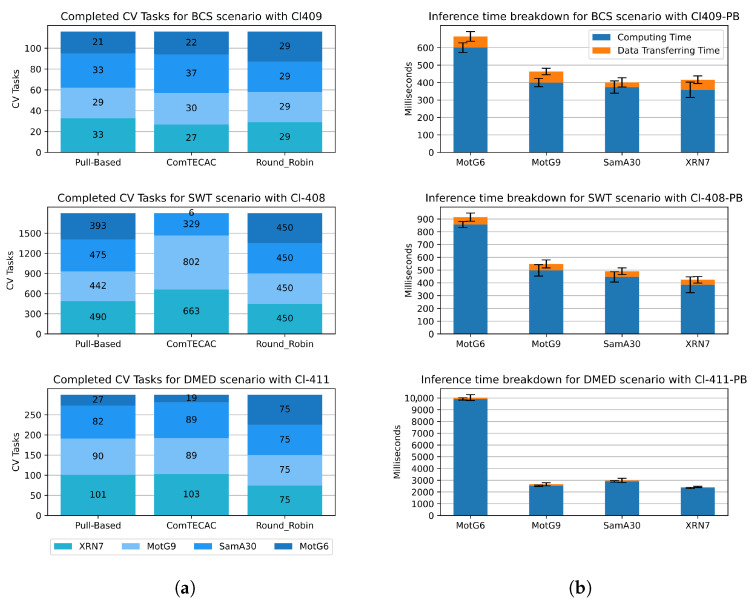
Insights of the most competitive smartphone-based setups with four nodes for the BCS, SWT, and DMED scenarios. MotG6 = Motorola Moto G6, SamA30 = Samsung A30, XRN7 = Xiaomi Redmi Note 7, MotG9 = Motorola Moto G9 Play. (**a**) Completed CV task breakdown by smartphone model with different load balancers. (**b**) Inference time breakdown per image for smartphone models in clusters using Pull-Based load balancer.

Figure 9 shows insights on how workload execution was distributed by different load balancing schemes among smartphones integrating the most competitive clusters with four nodes for all scenarios and a breakdown of the inference time registered by the mobile distributed inference approach showing the relations among computing/communication time disaggregated by smartphone models. Particularly, in Figure 9a, for all scenarios, it can be observed that, as expected with Round Robin load balancing, all smartphone models—differentiated in blue tones—are assigned with (and complete) the same amount of CV tasks. This behaviour does not allow for efficiently exploiting the computing heterogeneity of smartphones. With a Pull-Based scheme, on the contrary, smartphones are assigned new tasks as soon as they deliver the results of the previous pulled task. Such a reactive behaviour allows the distributed inference approach to assign CV tasks considering node heterogeneity. Notice that the XRN7 smartphone, which is one of the fastest within the clusters (see Figure 9b), is assigned the largest number of CV tasks in all scenarios. Conversely, MotG6, which is the slowest, is assigned the least CV tasks. Finally, ComTECAC CV task amounts are similar to those of the Pull-Based scheme, especially in short-duration workload scenarios like BCS and DME, which explains why both load balancing schemes achieved such similar accumulated inference latency values. Contrary to the Pull-Based scheme, ComTECAC assigns CV tasks proactively, i.e., deciding which node should be in charge of the task as soon as the task is created using a formula that combines network performance, node computing capability measured in FLOPS, queued tasks, and historic contributed energy measured through current battery level. For short-duration scenarios, few to none of these indicators vary—i.e., they remain almost constant. Consequently, each node’s rank is mostly decided by their FLOPS indicator value. This explanation does not apply to the behaviour that ComTECAC presents in the SWT scenario with a 30 min workload, where contributed energy varies. Certainly, this scenario provides evidence that the ComTECAC formula can be subject to further improvements. Lastly, Figure 9b shows how inference time is divided for individual images—not CV tasks—into computing time and data transferring time. Clearly, in all scenarios for all smartphone models, computing time dominates transferring time describing ratios that are in [1/13–1/19] for BCS, [1/10–1/15] for SWT and, [1/27–1/85] for DMED scenario. This could be taken as a hint of how energy consumption of the mobile distributed inference approach is disaggregated. Unless energy consumption in transferring time exceeds that of computing time by the inverse ratio of those reported, we could say that computing time dominates energy consumption.

#### 4.1.3. Battery Utilization Analysis

**Figure 10:**
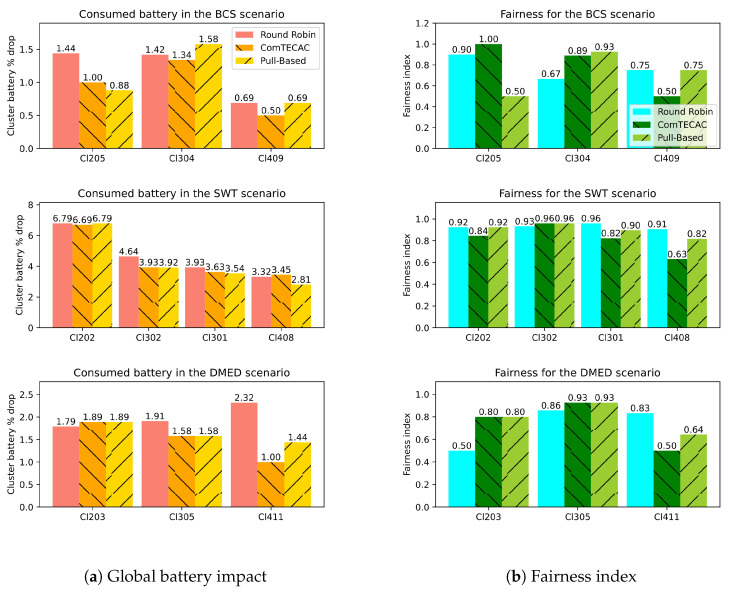
CV tasks impact on clusters global energy availability and Fairness index.

For smartphone-based setups, it is relevant to show the impact of CV task computations on cluster energy availability. Provided that clusters are composed of at least two nodes, cluster battery can be seen as the aggregation of node battery. To represent the cluster current battery level with a single positive integer value, we aggregate the Joules each smartphone contributes to the global cluster energy. By assuming that a cluster with a 100% global battery level is one where batteries of all integrating smartphones are fully charged, using Equation (1), it is possible to calculate the global battery level expressed as a percentage of a cluster for different smartphones’ battery levels. Figure 10a shows global battery level drops for all smartphone-based setups evaluated in all scenarios. Since in all cases smartphone-based setups run workloads while unplugged from the electricity grid, i.e., with batteries in discharging mode, the battery drops were calculated as the difference between the initial and the final global battery level of a cluster before and after the workload execution, respectively. In Figure 10a, it can be noted, for instance, that CV tasks in the SWT scenario causes the highest global battery drops compared to BCS and DMED, which is mainly due to the length of the workload execution ${-30}^{\prime }$ compared to ${∽4}^{\prime }$ and ${∽9}^{\prime }$ in BCS and DMED, respectively.

Another observation that emerges from Figure 10a is that when comparing cluster battery level drops between clusters w.r.t. the same scenario, they appear to decrease as cluster size increases. In fact, as more smartphones are integrated in a cluster, the cluster battery also increases and a percentage of this cluster battery is represented by a larger number in Joules. Something similar happens when two clusters are of the same size but one has smartphones whose batteries have more energy storage capacity than the other, which is the case for Cl301 and Cl302. However, a cluster with a larger battery does not imply that it will complete tasks in a more energy-efficient way. As an example, refer to the “Max. Cluster Energy” column of Table 4 to appreciate that Cl302 has slightly larger cluster battery capacity than Cl301, because the Xiaomi RN7 with a battery capacity of 4000 mAh in Cl301 was swapped with the Samsung A02 with a battery capacity of 4900 mAh in Cl302. However, according to Figure 10b, the latter smartphone is almost four times slower at performing a task in the SWT scenario than the former, meaning that Cl302 will spent more energy than Cl301 in completing the same number of tasks. This explains why, despite having a larger cluster battery, the battery drop of Cl302 is larger than that of Cl301 in the SWT scenario shown in Figure 10a. Moreover, it is relevant to point out that, in general, the Pull-Based load balancing scheme achieves equal or less global battery drops than Round Robin in approximately 0.5%. The ComTECAC heuristic was designed to consider energy contribution in combination with performance indicators to rank candidate nodes; however, it does not show a clear advantage over the Pull-Based scheme in this regard. In most cases, it causes similar global battery drops as the Pull-Based technique while a few others result in fewer battery drops.

Finally, we analyse fairness values derived from applying the Round Robin, ComTECAC, and the Pull-Based load balancing schemes. The index serves as a hint on how the edge cluster utilizes energy from all participating smartphones. The more the value approximates to one, the more balanced the battery utilization is among nodes, which, in turn, is an indicator of how well the level of parallelism is maintained over time. Figure 10b reveals that in some clusters, the Pull-Based scheme achieves slightly higher fairness values than those of Round Robin, while in other cases, the results are the opposite. There are cases where both schemes achieve equal fairness values. By contrast, ComTECAC fairness values are mostly under those obtained by the Pull-Based approach and in some cases, they are also under Round Robin fairness values. There is not a clear pattern to conclude that one load balancing scheme is better than the others according to the fairness index.

## 5. Discussion

### 5.1. Limitations

A few previous studies also focused on proving the strengths of edge computing for reducing end-to-end latency and allowing the execution of data-intensive IoT and delay-sensitive applications [17,45]. In this work, we explored how edge computing capabilities can be augmented with smartphone clusters under different workload scenarios for real-time AI inferences involving image streams. The competitive performance and reduced energy consumption that smartphone clusters showed, along with the pervasiveness of such devices worldwide, evidence their suitability for being considered a powerful tool to complement in-the-field, already present edge settings, and/or to fully support opportunistic distributed inference. Even though we showed through extensive experiments that it is feasible to achieve low-latency AI inferences for different DL-based resource-intensive workloads by exclusively relying on the computing power provided by smartphones clusters, we are aware of aspects that might negatively affect usability in real-world deployments and overall performance in exigent generic application contexts. One aspect is the impact of network conditions, specifically variability in smartphones’ signal strength, which could increase the retransmission rate of packets and the available bandwidth that other user processes and applications could share with the collaborative inference software. User processes and applications might also introduce uncontrolled variability in the usage of computing resources available to make inferences. Furthermore, in the case of interactive applications, the user might experience faster battery drops due to the screen usage. The experiments performed considered smartphones connected with excellent signal strength to a 5 GHz WiFi network; they also entailed restricted network activity of user applications other than the collaborative inference software and guaranteed that smartphone screens stayed off while inferences were calculated. It is within our roadmap to incorporate new features in the experimentation toolset to allow us and other researchers to emulate and control resource variability aspects and, ultimately, cover more experimental scenarios.

In relation to pre-trained AI models, to assure homogeneity in result quality, it is necessary to perform smartphone model synchronization. This applies especially to those subject to frequent updates by maintainers. This, in turn, might introduce undesired delay at the beginning of a distributed inference session. This aspect is relevant to the implementation of desirable middleware features.

With respect to implementation details, we also want to mention possible technical improvements to the cluster management as well as intra-device resource usage that could bring out other latency–energy consumption tradeoffs. One of these is the support of different task distribution methods. Currently, collaborating smartphones in the cluster request the master node for task metadata using HTTP messages to first load into memory the DL model to perform inferences. Then, smartphones request input (i.e., tasks) through new HTTP requests. Varying the ways in which master and collaborating nodes communicate, e.g., with a connection persistent protocol like web sockets, would help to speed up the inference performance. Another improvement is to provide smartphones with the ability to dynamically vary the level of concurrency when executing tasks. This would allow collaborating nodes to regulate load—for instance, freeing resources to anticipate user interactions and battery overheating—or, on the contrary, offer more computing resources when the phone is idle. Current intra-device implementation supports the execution of at most one task.

Finally, the results obtained demonstrate that low-latency and energy efficient solutions are achievable with the DL models used for the described workload scenarios. However, trying other DL modes architectures or model versions resulting from the application of size reduction techniques (e.g., quantization, pruning, low-rank factorization, etc.) might signify a boost in energy utilization and latency but falls out of the scope of this work. It is worth mentioning that comparisons involving different model versions or model architectures for a specific problem are commonly carried out considering the precision of results or other metrics that allow for differentiating not only latency but also the quality of results. In our results, quality was not within the dimension of analysis because all setups studied for each workload scenario ran the same model.

### 5.2. Future Work

Several questions remain open, demanding further research. One of these relates to whether current software stacks including middlewares for distributed computing [46,47] and frameworks for AI execution [48] in low-powered devices are prepared to integrate smartphone clusters as a new type of edge node by attending diverse aspects related to computational resource provision with devices that present non-dedicated resources—i.e., with user applications that must prioritize the usage of resources and deal with unstable communication due to physical position changes of device owners, limitations in resource scavenging times due to battery operation times, and compute-while-charging scenarios where computations are performed by battery-powered devices while plugged to the electricity grid [49] or while harvesting energy using radio-frequency signals of access points [16].

Another open question concerns incentives for making device owners offer up unused computing mobile resources. The literature describes at least two approaches. One of them is associated with altruistic attitudes of devices owners—this is similar to what is promoted in volunteer computing platforms with unused computing cycles of desktop computers for scientific projects. Applications include daily normal situations that require in-the-field inference capabilities either for reducing the cost of data transmission—e.g., chronic disease health monitoring, safety-guard mechanisms in risky situations (e.g., alerting about obstacles in the road while driving), providing real-time responses to improve user experience (e.g., augmented reality applications in tourism-related activities), etc. By contrast, the other incentive approach we envision is based on opportunistic inferences as a service approach that benefits device owners with some revenue in the form of credits [50], reputation [51,52], or energy [16] in exchange for completing tasks that require in-the-field inference capabilities—for example, as a complement to surveillance cameras for crime prevention in public spaces. The obtained results achieved in this study with efficient hardware, such as that embedded in smartphones, w.r.t using other edge nodes are promising in terms of energy consumption and performance. However, massive application of the approach must not be carried out without informing users about the side effects regarding quicker losses in device battery life as a consequence of more intensive use of devices’ computational nd communication resources, which in turn can result in excessive electronic waste generation, especially when using devices with non-replaceable batteries or when device replacement is encouraged rather than simply replacing the battery.

In this regard, another aspect worth mentioning that is intimately related to AI and computing infrastructures concerns energy consumption and, hence, indirectly, CO_2_ emissions. In its World Energy Outlook 2024 [53,54], the International Energy Agency (IEA) projects that energy consumption in datacenters is set to expand in the future, in part due to the digitalization of the economy but also as a consequence of the rapid advance of AI technologies and the outbreak of AI startups. In fact, figures from 2022 also reported by the IEA—i.e., some years before the AI boom we have been experiencing since then—indicated that datacenters consume 1–2% of the world’s electricity, which is backed up by estimates from similar studies [55,56]. This positions energy-efficient approaches to edge computing as a crucial path to cap these estimates while providing viable computing settings to continue helping bringing AI to the masses. Edge computing research should not only merely quantify energy savings compared to traditional computing approaches but also develop mechanisms to measure the impact on ${\mathrm{CO}}_{2}$ emissions at the software level, such as in [57]. Quantification must also consider current mobile hardware manufacturing practices, for example, promoting replaceable (sealed) batteries, which encourage users to upgrade to a new device when battery performance declines, rather than simply replacing the battery. Metrics such as the Software Carbon Intensity (SCI) index [58] might serve as a pertinent reference since it captures the environmental impact of software in terms of ${\mathrm{CO}}_{2}$ emissions considering the carbon intensity of the energy used to run software and the embedded emissions due to the carbon footprint to build the hardware on which the software runs.

There are also improvement opportunities in line with developing new load balancing heuristics, and based on the results from preliminary runs, we plan to improve previously published heuristics that use MFLOPs to rank node computing capabilities [39]. An alternative solution is to use information derived from the Tensorflow benchmarking tool [33]. In spite of differences observed in inference times obtained via the Tensorflow benchmarking tool and results collected in experiments using real inputs, relative benchmarked positions between edge nodes are similar, meaning that it is feasible to use such a tool to build node rankings that can serve as input for the load balancing component to distribute tasks based on an alternative node performance score to the classic MFLOPs.

Another future research direction is to consider other forms of workload distributions, i.e., that exploit the structure of deep learning (DL) architectures—for example, split computing, early exiting techniques, and model quantization [59]—to better exploit the CPU capabilities of mobile platforms while performing network inferences.

## 6. Conclusions

In this work, we explored the computing capabilities of mobile distributed computing setups for real-time inferences by experimenting with heterogeneous AI workloads, built upon real convolutional neural network (CNN) models and image datasets that were used to emulate computer vision (CV) tasks generated in a stream-like fashion. We ran and compared the performance achieved by several setups built with commodity hardware including single-board computers (SBCs) and smartphone clusters that can be found or exploited in practical edge computing and IoT application scenarios.

We conclude that tiny clusters composed by two to four low-to-middle-end smartphones using a Pull-Based load balancing scheme offer competitive performance compared to an SBC equipped with a reasonable CPU (Intel core i5 8th generation) in computing real-time CV inference tasks. This suggests that to satisfy certain delay-sensitive application scenarios, the exploitation of already-in-place smartphones clusters can be considered before the investment and deployment of SBCs. According to Table 3, the necessary investment for supporting the evaluated scenarios using a powerful SBC like the Gigabyte Brix is only 30% cheaper than with a cluster-based setup comprising four low-to-mid-end smartphones. This is when including hardware acquisition cost of smartphones. By contrast, if computing capability is hired out to smartphones’ owners already present in the application context, then the equation of computing resources cost can be different. In any case, energy consumption measurements show that the tradeoff including performance and energy consumption favours smartphone clusters over SBCs. The former can deliver competitive performance with a reduction of around three times the energy consumption required by a powerful SBC such as the Gigabyte Brix.

## Figures and Tables

**Table 1:** Schemes for distributed AI inference.

Distributed Inference Scheme	Associated Works
Vertical	[17,18,19,20,21]
Horizontal	[22,23,24,25,26]

**Table 2:** Approaches for horizontal distributed AI inference at the edge.

Parallelism Type	Experiments		Online Adaptive QoS Support?	Main Motivation	Work
	Resource Heterogeneity Type	Type			
Model Partitioning	Homogeneous nodes	Real Hardware	No	Automate large CNN model partitioning, deployment, and execution on multiple resource-constrained edge devices	[22]
	Homogeneous nodes	Real Hardware	Yes	Accelerate performance with an intra-layer parallelism instead of layers sequential execution as in traditional hierarchical inference	[23]
Data partitioning	Heterogeneous nodes	Real Hardware	Yes	Exploit the concept of approximate computing to ensure performance and accuracy constraints considering heterogeneity of the edge cluster and node availability	[24]
	Heterogeneous nodes	Real Hardware	Yes	Compare inference delivery capabilities and energy efficiency of IoT devices versus clusters of smartphones	This work
Hybrid	Homogeneous nodes	Real Hardware	Yes	Allow large DL models to execute in resource-limited devices achieving low memory footprint and execution latency	[25]
	Heterogeneous nodes	Simulation and real hardware	Yes	Reduce mobile edge computing server’s computing pressure	[26]

**Table 3:** Edge computing nodes: hardware features.

Edge Node Type	Edge Node Name	Short Name	Processor	RAM (GB)	Conectivity	Battery Capacity (mAh)	Avg. Price (USD)
SBC	Raspberry Pi 4	RPi4	Quad-core 1.5 GHz ARM Cortex-A72	4	Wireless	N/A	55
	Nvidia Jetson Nano	NvJN	Quad-core ARM Cortex-A57 MPCore	4	Wired	N/A	99
	Gigabyte Brix GB-BRi5H-8250	GBx	Quad core i5-8250U	8	Wireless	N/A	500
Smartphone	Samsung A02	SamA02	Quad-core 1.5 GHz Cortex-A53	2	Wireless	4900	116
	Motorola Moto G6	MotG6	Octa-core 1.8 GHz Cortex-A53	3	Wireless	3000	160
	Samsung A30	SamA30	Octa-core (2 × 1.8 GHz Cortex-A73 & 6 × 1.6 GHz Cortex-A53)	3	Wireless	3900	170
	Xiaomi Redmi Note 7	XRN7	Octa-core (4 × 2.2 GHz Kryo 260 Gold & 4 × 1.8 GHz Kryo 260 Silver)	4	Wireless	4000	200
	Motorola Moto G9 Play	MotG9	Octa-core (4 × 2.0 GHz Kryo 260 Gold & 4 × 1.8 GHz Kryo 260 Silver)	4	Wireless	5000	200

**Table 4:** Details of smartphone-based setups.

Cluster ID	Cluster Size	Smartphone Models	Smartphones Initial Battery %	Cluster Initial Energy (Joules)	Cluster Max. Energy (Joules) ^1^	Cluster Initial Battery % ^2^
Cl202	2	XRN7, MotG9	87, 75	99,532.80	123,840	80.37
Cl203	2	XRN7, MotG9	27, 54	51,904.80	123,840	41.91
Cl205	2	MotG9, SamA30	54, 30	53,152.20	122,454	43.41
Cl301	3	SamA30, XRN7, MotG9	27, 45, 60	80,582.58	177,894	45.30
Cl302	3	SamA30, SamA02, MotG9	27, 45, 60	86,195.88	190,368	45.28
Cl304	3	MotG6, XRN7, MotG9	77, 25, 53	81,712.80	164,880	49.55
Cl305	3	MotG9, MotG6, SamA30	54, 92, 25	88,206.30	163,494	53.95
Cl408	4	MotG6, SamA30, XRN7, MotG9	30, 27, 45, 60	92,894.58	218,934	42.43
Cl409	4	MotG6, SamA30, XRN7, MotG9	88, 80, 38, 49	133,941.6	218,934	61.18
Cl411	4	MotG6, SamA30, XRN7, MotG9	25, 52, 44, 38	88,753.68	218,934	40.54

^1^ To calculate this value, we used a formula derived from Formula (1) assuming each smartphone is fully charged. ^2^ The value was calculated using Formula (1), replacing batteryDrops by the battery level of each smartphone at the beginning of a workload test.

## Data Availability

The original contributions presented in this study are included in the article. Further inquiries can be directed to matias.hirsch@isistan.unicen.edu.ar.

## Appendix A

Table A1, Table A2 and Table A3 contain the data reported in graphs presented in Section 4.1 for the BCS, SWT, and DMED scenarios.

**Table A1:** Body Condition Score scenario (03:50 min data stream).

Edge Setup		Load Balancing	Acc. Inference Latency (s)	Jain’s Fairness Index	Cluster Consumed Battery (%)	Consumed Energy (Joules)
SBC-based	RPi4	N/A	1467	N/A	N/A	3501.97
	NvJN	N/A	134.4	N/A	N/A	2147.13
	GBx	N/A	3.0	N/A	N/A	4524.82
Smartphone-based	Cluster 205	Round Robin	251.4	0.9	1.44	1765.08
		ComTECAC	422.4	1.0	1.0	1224.54
		Pull Based	181.7	0.5	0.88	1081.08
	Cluster 304	Round Robin	181.8	0.667	1.42	2341.29
		ComTECAC	141.6	0.889	1.34	2203.2
		Pull Based	71.4	0.926	1.58	2605.10
	Cluster 409	Round Robin	76.2	0.750	0.69	1510.64
		ComTECAC	16.9	0.5	0.5	1094.94
		Pull Based	10.8	0.750	0.69	1510.64

**Table A2:** Sense while Travel scenario (30 min data stream).

Edge Setup		Load Balancing	Acc. Inference Latency (s)	Jain’s Fairness Index	Cluster Consumed Battery (%)	Consumed Energy (Joules)
SBC-based	RPi4	N/A	2094.6	N/A	N/A	14,827.11
	NvJN	N/A	282.6	N/A	N/A	10,246.13
	GBx	N/A	1.2	N/A	N/A	22,403.73
Smartphone-based	Cluster 202	Round Robin	903.6	0.924	6.79	8408.74
		ComTECAC	858.6	0.845	6.69	8280.00
		Pull Based	869.4	0.924	6.79	8408.74
	Cluster 302	Round Robin	678.0	0.933	4.64	8833.07
		ComTECAC	311.9	0.960	3.93	7476.12
		Pull Based	131.4	0.960	3.92	7462.43
	Cluster 301	Round Robin	31.2	0.960	3.93	6991.23
		ComTECAC	21.1	0.823	3.63	6459.48
		Pull Based	16.2	0.896	3.54	6297.45
	Cluster 408	Round Robin	10.8	0.907	3.32	7268.61
		ComTECAC	21.1	0.631	3.45	7554.42
		Pull Based	6.0	0.818	2.81	6152.04

**Table A3:** Disease Monitoring and Early Diagnosis scenario (08:55 min data stream).

Edge Setup		Load Balancing	Acc. Inference Latency (s)	Jain’s Fairness Index	Cluster Consumed Battery (%)	Consumed Energy (Joules)
SBC-based	RPi4	N/A	1345.8	N/A	N/A	6302.99
	NvJN	N/A	445.8	N/A	N/A	3733.47
	GBx	N/A	3.6	N/A	N/A	7834.28
Smartphone-based	Cluster 203	Round Robin	351.56	0.5	1.79	2217.60
		ComTECAC	421.72	0.8	1.89	2347.20
		Pull Based	301.56	0.8	1.89	2347.20
	Cluster 305	Round Robin	1485.0	0.857	1.91	3122.73
		ComTECAC	607.71	0.926	1.58	2583.20
		Pull Based	236.4	0.926	1.58	2583.20
	Cluster 411	Round Robin	978.6	0.833	2.32	5079.27
		ComTECAC	36.12	0.5	1.0	2189.88
		Pull Based	25.8	0.643	1.44	3152.65
